# Assessment of Color Stability of Various Flowable Composite Resins with Different Viscosities

**DOI:** 10.3390/biomimetics10080550

**Published:** 2025-08-21

**Authors:** Gülşah Yenier Yurdagüven

**Affiliations:** Department of Restorative Dentistry, Faculty of Dentistry, Istanbul Okan University, İstanbul 34959, Türkiye; gulsahyenier@gmail.com

**Keywords:** composite resins, color stability, staining agents, spectrophotometry, viscosity, repolishing, aesthetic dentistry, optical properties

## Abstract

Biomimetic restorative dentistry aims to preserve tooth structure and achieve optimal aesthetic harmony with surrounding dentition. The principles and protocols associated with biomimetic restorative dentistry are designed to enhance the longevity of the restoration. The use of flowable CRs is increasingly common; however, the effect of viscosity on the discoloration has not been clearly established. This in vitro study aimed to assess the color stability of flowable CRs with varying viscosities following immersion in common staining solutions and subsequent repolishing. A total of 250 disc-shaped specimens (8 mm × 2 mm) were prepared from five CRs with different viscosity profiles: high-viscosity (Spectra STHV, Dentsply, Milford, DE, USA), medium-viscosity (Estelite Universal Flow Medium, Tokuyama Dental Co., Tokyo, Japan), bulk-fill (Estelite Bulk-Fill Flow, Tokuyama Dental Co., Tokyo, Japan; SDR Plus, Dentsply, Milford, DE, USA), and packable (Estelite Posterior, Tokuyama Dental Co., Tokyo, Japan). After polymerization and baseline color measurements, specimens were immersed in coffee, tea, cola, red wine, or distilled water for 144 h. Color values were recorded before and after staining, and again following repolishing. Color changes (ΔE_1_, ΔE_2_, ΔE_3_) were calculated using the CIE Lab system and statistically analyzed via two-way ANOVA and Tukey HSD (α = 0.05). Both the CR type and the staining solution substantially affected the color change. SDR Plus exhibited the highest ΔE values. Red wine caused the most discoloration. Repolishing enhanced color in selected groups.

## 1. Introduction

The term “*biomimetic*” is derived from the Latin word “*bio*”, signifying life, and “*mimetic*”, which pertains to the imitation of biochemical processes inspired by natural phenomena [1]. The main goal of biomimetic restorative dentistry is the reestablishment of the integrity and functionality of dental hard tissues through restorative materials that replicate the tooth’s biomechanics, structural integrity, and aesthetic harmony [2]. This approach emphasizes the maximal preservation of dental tissues through the use of restorative materials that closely mimic the properties of natural structures [3]. To achieve these outcomes, biomimetic restorative protocols are generally divided into two complementary categories: stress-reducing protocols and bond-maximizing protocols [4]. The biomimetic concept is of paramount importance in the advancement of restorative dental materials and enhancing the field of adhesive dentistry [5].

Composite resins (CRs) have significantly contributed to this philosophy by enabling minimally invasive procedures that maintain healthy tooth structure while delivering natural-looking esthetic outcomes [6]. In response to evolving clinical demands, manufacturers have introduced CRs specifically designed with a wide range of formulations tailored for diverse indications. Each formulation is characterized by unique combinations of resin matrix and filler content [7]. The resin matrix composition is exclusively derived from methacrylate chemistry, incorporating components such as BisGMA (bisphenol A glycidyl dimethacrylate), TEGDMA (triethylene glycol dimethacrylate), BisEMA (ethoxylated bisphenol A dimethacrylate), and UDMA (urethane dimethacrylate) [8]. The structure of the base monomer significantly influences the degree of conversion, water sorption, solubility, and color stability [9].

Over the past two decades, innovations in filler systems have led to major improvements in CR performance. Studies have highlighted how filler types—such as silica and glass—shape (e.g., spherical vs. irregular), and relative proportion critically affect mechanical properties and optical behavior [10,11]. Reduced filler particle size, transitioning from macrofillers to nano-hybrids, has notably enhanced polishability, wear resistance, and translucency [12]. These advances have also contributed to favorable changes in viscosity, thereby improving material handling [13,14,15,16].

Viscosity plays a pivotal role in determining the clinical behavior of CRs, directly affecting their adaptability, application technique, and mechanical performance [17]. Flowable CRs provide better cavity adaptation and wettability, though earlier versions were mechanically inferior due to their lower filler content, greater polymerization shrinkage, and insufficient wear resistance [17,18]. Previously, they were mainly used in areas of minimal stress, often as bases or liners [19,20]. Recent advances have significantly improved the mechanical integrity of flowable CRs, expanding their use in a broader range of clinical scenarios beyond traditional indications [11].

High-viscosity CRs are often preferred for their superior mechanical strength, increased filler loading, enhanced wear resistance, reduced polymerization shrinkage, and favorable sculptability [11,14]. However, their limited flowability may impede adaptation to internal cavity walls. To overcome these limitations, low-viscosity flowable CRs are frequently utilized. These materials provide improved wettability, facilitating better adaptation and reducing the need for manual manipulation during placement [11]. Moreover, they mitigate the risk of air entrapment and the occurrence of void formation [21].

One major innovation is the introduction of bulk-fill flowable CRs, which streamline restorations by allowing placement in thicker increments and considerably reduce the chair time required for clinicians [22,23]. However, due to their lower filler content, these materials typically require a final occlusal capping layer of more highly filled CR to enhance resistance under occlusal loading [24,25].

Despite these advancements in the formulations, CRs remain susceptible to discoloration over time, which may result from both intrinsic and extrinsic factors [26]. This susceptibility underscores the clinical importance of maintaining color stability, as the color match is a key parameter in widely adopted FDI World Dental Federation criteria for restorations [27]. Intrinsic factors pertain to the chemical composition and structural integrity of the resin itself. These include the oxidation of unpolymerized monomers and amine accelerators, which can compromise the stability of chemical bonds [26,28]. Moreover, filler characteristics—such as their content, particle size distribution, filler/matrix proportion, and surface treatment—also contribute significantly to the aesthetic performance and long-term color stability of CRs [29]. The type of photo-initiator also influences the resin’s susceptibility to color changes [30]. Extrinsic factors, on the other hand, relate to the accumulation of dental plaque, the consumption of certain foods and beverages, particularly those that are highly pigmented (such as coffee, red wine, and tea), and the effects of tobacco use, which can lead to staining of the CR over time [26,31]. Additionally, surface roughness and structural irregularities exacerbate pigment retention and discoloration [32]. Discoloration of tooth-colored CRs often necessitates restoration replacement, introducing time and cost burdens for both practitioners and patients [33].

Biomimetic restorative dentistry focuses on restoring and replicating the natural tooth’s biomechanics, structural integrity, and esthetic harmony through the use of advanced restorative materials [2,4,5]. Numerous studies have investigated staining effects on CRs [34,35,36,37,38,39,40,41,42,43]. However, the existing literature continues to present conflicting findings- particularly regarding materials with different viscosities [13,36,37,38,39,40,41,42,43]. As the use of flowable CRs gains popularity, their performance in aesthetically demanding contexts requires further scrutiny, and the relationship between their viscosity and discoloration, as well as recovery following repolishing, has yet to be definitively clarified. This in vitro study aimed to evaluate the color stability of flowable CRs with varying viscosities after immersion in commonly consumed staining solutions and subsequent repolishing. The null hypotheses tested were as follows: (i) staining solutions would not induce a significant color change in the CRs, and (ii) the repolishing procedure would significantly affect their color values.

## 2. Materials and Methods

### 2.1. Specimen Preparation

Table 1 presents the CR materials used in this study: high-viscosity flowable CR (Spectra STHV (SSTHV), Dentsply), medium-viscosity flowable CR (Estelite Universal Flow Medium (EUFM), Tokuyama), bulk-fill CRs (Estelite Bulk-Fill Flow (EBF), Tokuyama; SDR Plus (SDRP), Dentsply), and packable CR (Estelite Posterior (EP), Tokuyama).

The sample size was determined a priori using the G*Power v3.1.9.7. software (Düsseldorf, Germany) program based on effect sizes from a previous study [41]. For α = 0.05 and power (1 − β) = 0.80, the required sample size *n* = 10 specimens per subgroup (total N = 250) was included to ensure adequate power.

A fifty-disc sample of each CR, measuring 2 mm in height and 8 mm in diameter, was fabricated using standardized silicone ring molds. The molds, placed on glass plates, were filled with CR and subsequently covered with a Mylar strip (Kerr, Orange, CA, USA). The assembly was slightly compressed using additional glass plates, and light polymerization was performed according to the manufacturer’s instructions. The light-curing unit (Elipar Deep-Cure S10, Solventum, St. Paul, MN, USA) was positioned perpendicularly at a fixed distance of 1 mm from the specimen surface. Following polymerization, the specimens were finished and polished using a sequential series of Sof-Lex discs (Sof-Lex; Solventum, 3 M Deutschland GmbH, Neuss, Germany) in decreasing grit order. Each disc was applied for 20 s with a low-speed handpiece operating at 5000 rpm. After each polishing step, the specimens were thoroughly rinsed under running water for 10 s. A new disc was used for each specimen. Subsequently, all samples were stored in distilled water at 37 °C for 24 h prior to baseline color measurement (T0).

### 2.2. Staining Procedures

Following baseline color measurements, specimens from each composite resin (CR) group were randomly divided into five subgroups (*n* = 10), each assigned to one of the following immersion media: red wine, coffee, tea, cola, or distilled water.

- Red Wine Group: Specimens were immersed in 50 mL of red wine (Shiraz, Doluca, Türkiye; pH = 3.50).
- Coffee Group: A coffee solution was prepared by dissolving 3.6 g of instant coffee (Nescafe Classic, Nestlé Türkiye Gıda, Istanbul) in 300 mL of boiling distilled water. Specimens were immersed in 50 mL of this solution (pH = 4.50).
- Tea Group: Black tea solution was prepared by steeping one tea bag (Tek Dem Classic, Lipton, Istanbul, Türkiye) in 100 mL of hot water (approximately 95 °C) for 2 min. Each specimen was immersed in 50 mL of freshly prepared tea (pH = 5.30).
- Cola Group: Specimens were immersed in 50 mL of cola (Pepsi, PepsiCo, Istanbul, Türkiye). To maintain consistency, a freshly opened bottle was used daily (pH = 2.53).
- Distilled Water Group (Control): Specimens were stored in 50 mL of distilled water (pH = 7.55) and served as the control group.

All specimens were fully submerged in their respective solutions in sealed containers and incubated at 37 °C for 144 h. Solutions were renewed every 24 h to prevent microbial growth and preserve the chemical properties of the staining agents. Upon completion of the staining period, color measurements were repeated to obtain post-staining values (T1).

### 2.3. Repolishing Procedure

Following the staining period, all specimens were thoroughly rinsed with distilled water and gently dried using paper towels. Color measurements were then conducted to assess discoloration post-immersion. Subsequently, each specimen underwent a standardized repolishing protocol using Sof-Lex discs (Solventum, 3 M Deutschland GmbH, Neuss, Germany). Medium grain (2381M), fine grain (2381F), and superfine grain (2381SF) discs were applied for 20 s per surface at 5000 rpm, using a low-speed handpiece and without water cooling. To ensure procedural uniformity, a new polishing disc was used for each specimen. Upon completion of the repolishing procedure, final color measurements were performed again (T2).

### 2.4. Color Measurements

Color measurements were conducted at three distinct time points: baseline (T0), after staining (T1), and following the repolishing procedure (T2). A clinical spectrophotometer (VITA Easyshade Advance 4.0; Vita Zahnfabrik, Bad Säckingen, Germany) was calibrated prior to each session according to the manufacturer’s instructions. All measurements were performed against a standardized gray background under consistent ambient lighting conditions. Each specimen was measured three times, and the mean value was used to obtain the L*, a*, and b* coordinates according to the CIE Lab* color system. In this system, L* represents lightness ranging from black (0) to white (100), a* indicates the green (−) to red (+) spectrum, and b* denotes the blue (−) to yellow (+) spectrum.

The overall color change (ΔE_ab_ between time points was calculated using the following formula:

ΔE_ab_ = √[(ΔL*)^2^ + (Δa*)^2^ + (Δb*)^2^]

ΔE_1_ = T1 − T0, ΔE_2_ = T2 − T1, and ΔE_3_ = T2 − T0

Based on previously established thresholds in the literature, color changes greater than ΔE > 3.3 are considered clinically unacceptable [31].

### 2.5. Statistical Analysis

Statistical analyses were carried out using IBM SPSS Statistics, version 22.0 (IBM Corp., Armonk, NY, USA). The normality of the data distribution was assessed through the Kolmogorov–Smirnov and Shapiro–Wilk tests. Upon confirming that the data followed a normal distribution, a two-way ANOVA was conducted, along with post hoc Tukey tests, to evaluate the combined effects of the CR and immersing solution. A *p*-value of less than 0.05 was determined to be statistically significant.

## 3. Results

In all three assessed ΔE parameters (ΔE_1_, ΔE_2_, and ΔE_3_), statistically significant differences were observed among the CRs (*p* = 0.001), among the immersing solutions (*p* = 0.001), and for the interaction between CR and solution (*p* = 0.001) (Table 2).

### 3.1. ΔE_1_ Findings

According to ΔE_1_ scores, SDRP exhibited the highest level of discoloration in coffee, tea, and cola solutions compared to all other CRs (*p* < 0.001), exceeding the clinically acceptable threshold (≥3.3). No statistically significant differences were found among the remaining CRs. Color changes in CRs, aside from SDRP, were acceptable for tea and cola solutions but not for coffee. Additionally, regardless of the CR used, samples immersed in red wine resulted in the most severe and clinically unacceptable discoloration (*p* < 0.001) compared to the other solutions. Among all CRs immersed in water, color changes remained within acceptable limits, with EUFM demonstrating the lowest ΔE_1_ value (Table 3).

### 3.2. ΔE_2_ Findings

According to the ∆E_2_ scores, SDRP demonstrated significantly higher color change compared to the other CRs in all solutions, with the exception of wine. Within the wine group, EP exhibited the greatest color change, while no notable differences were detected among the remaining CRs. The samples of SDRP displayed unacceptable ∆E_2_ values in all solutions, though a significant difference was observed only between coffee and water (*p* < 0.05). Furthermore, the ∆E_2_ values of SSTHV and EP samples immersed in wine were markedly higher than those in other solutions. EUFM and EBF, while showing increased ΔE_2_ values, remained significantly more stable in tea, cola, and water solutions (Table 4).

### 3.3. ΔE_3_ Findings

Regarding ΔE_3_ values, SDRP exhibited the highest discoloration in both cola and water solutions compared to the other CRs. It also recorded the greatest ΔE_3_ in coffee among all tested CRs, with the exception of EBF. In tea, the SDRP again showed the greatest ΔE_3_ value with the exception of EUFM, which had comparable values. Notably, the EP achieved the most substantial ΔE_3_ value among all tested CRs. For wine immersion, all CRs demonstrated statistically higher ΔE_3_ values than in any other solution. In coffee, EBF and SDRP both showed significantly greater discoloration than in tea, cola, or water, whereas no significant inter-solution differences were observed in the remaining CRs (Table 5).

## 4. Discussion

This in vitro study evaluated the color stability of various flowable CRs with differing viscosities after immersion in colorant solutions. The findings revealed that specimens immersed in red wine exhibited the most pronounced and clinically unacceptable discoloration. For the other staining media, the extent of discoloration varied depending on the CR, corroborating results reported in previous studies [36,37,38,39,40,41,43]. Moreover, the repolishing procedure performed after staining influenced color recovery to a certain extent, suggesting that surface treatment may partially reverse extrinsic discoloration. Based on these findings, the null hypothesis—stating that CRs would not undergo significant color change following exposure to staining agents and subsequent repolishing would significantly affect their color values—was rejected.

When CR materials are exposed to liquids, the majority of water absorption by the organic matrix occurs within the first four days, with peak uptake typically observed within the first week [44,45]. In alignment with these findings, the present study employed a 144 h immersion period, consistent with the widely accepted timeframe reported in previous literature [31,41]. This extended exposure duration is considered to simulate approximately six months of daily intake of chromogenic beverages under in vivo conditions [31].

The literature indicates that various formulae have been utilized to assess color changes in dental materials, including ΔE_ab_, ΔE_00_, and combinations thereof. A comprehensive review identified ΔE_ab_ as the most frequently used method, reported in 158 studies, while ΔE_00_ appeared in 17 studies [26]. This study employed the widely accepted CIELAB color space and utilized the ΔE_ab_ formula, aligning with prevailing trends in the literature. Although ΔE_00_ provides improved perceptual uniformity, ΔE_ab_ was preferred due to its broad usage, ease of calculation, and compatibility with existing data. Accordingly, a clinically acceptable threshold value of ΔE ≤ 3.3 was adopted in this study [31].

Bulk-fill CRs have gained popularity in restorative dentistry due to their simplified application protocols and efficiency in reducing operative time [22]. However, concerns persist regarding their long-term aesthetic performance, particularly in terms of color stability [38,39,40,43]. In the present study, two bulk-fill composites, EBF and SDRP, were evaluated, the latter being a low-viscosity flowable CR. It is well known that low-viscosity bulk-fill materials typically require a final capping layer of conventional CR to enhance mechanical and aesthetic properties [8,25]. Notably, SDRP, according to the manufacturer, can be used without an additional capping layer in Class III and Class V restorations [46]. Nevertheless, our results demonstrated that SDRP exhibited the highest degree of discoloration among the tested CRs across all evaluation intervals (ΔE_1_, ΔE_2_, ΔE_3_). These findings align with previous reports indicating that low-viscosity bulk-fill materials are more prone to discoloration [36,40]. Based on these results, the use of SDRP without a capping layer in aesthetically demanding regions should be approached with caution.

To further elucidate the factors contributing to SDRP’s pronounced discoloration, the filler composition of the tested CRs was analyzed. Although EBF, SDRP, and EUFM exhibited similar filler volumes (70%, 70.5%, and 71%, respectively), their filler content by weight varied considerably: EUFM and EBF contained 57% and 56% by weight, respectively, while SDRP contained only 47.4%. This lower filler loading in SDRP implies a higher proportion of resin matrix, which increases water sorption and pigment uptake—both compromising color stability [47,48]. The findings presented in this study are consistent with prior studies linking lower filler content to diminished discoloration resistance in CRs [29,49].

Our findings are largely consistent with previous research; however, some discrepancies were noted when compared to the existing literature. For instance, Korkut and Hacıali reported that the CR exhibiting the highest discoloration was not the one with the lowest filler content (64.5% by weight and 42% by volume), but rather a regular-viscosity CRl containing 65% by weight and 46% by volume [41]. This result was attributed to the absence of UDMA in the resin matrix, which is a monomer recognized for its stain resistance and low water sorption [31]. In an earlier study, the water sorption capacities of common dental monomers were ranked in ascending order as follows: Bis-EMA, UDMA, Bis-GMA, and TEGDMA [16]. The present study evaluated various CRs, with the majority containing Bis-GMA, TEGDMA, and UDMA, as detailed in Table 1. While these monomers are characterized as the most hydrophilic, they did not emerge as the primary factors influencing discoloration in our study. It is noteworthy that the SDRP demonstrated the highest degree of discoloration across all time intervals, which can be correlated with its lower filler content. This research underscores the significant role that the characteristics of fillers play in color stability. Specifically, fillers help reduce the free volume within polymer matrices, thereby diminishing material sorption. As noted by Bociong et al., low filler content CRs exhibit the highest water absorption [50].

Additionally, the role of translucency should also be considered alongside filler composition [15]. A preceding study indicated that the mean translucency parameter values of flowable CRs exceeded those of their corresponding universal CRs, thereby underscoring an inverse relationship between filler content and translucency [13,15]. This variance in translucency between flowable and universal CRs has also been demonstrated to impact the resulting color differences between these materials [36]. These findings are relevant to the present study, SDRP—a low-viscosity bulk-fill material with reduced filler loading—exhibited the highest ΔE values across all time intervals. This elevated translucency may have increased light transmission and enhanced the visibility of intrinsic color changes caused by pigment uptake and water sorption, particularly in the absence of a capping layer.

Remarkably, EUFM showed the least discoloration after water storage, despite containing all the monomers associated with hydrophilic behavior. This superior performance may be attributed to its matrix composition. A preceding study corroborates this finding, indicating that the partial substitution of TEGDMA with UDMA comonomer in Bis-GMA/TEGDMA systems leads to a reduction in water absorption and an enhancement in color stability [51]. In contrast, even after repolishing, the SDRP group demonstrated that the ΔE_3_ value (T2–T0) following water immersion remained above the clinically acceptable threshold (4.86 ± 1.65). Notably, in the context of SDRP, the base monomer Bis-GMA has been entirely substituted with less viscous dimethacrylates, including UDMA and its derivatives. This change may contribute to the observed issues with color stability, as these materials are known to yield polymers that exhibit greater flexibility in comparison to Bis-GMA [52].

Nonetheless, the present results indicated that viscosity alone is not a definitive predictor of discoloration. High-viscosity and medium-viscosity flowable CRs, one of the bulk-fill CRs (EBF), and the packable CR exhibited statistically similar levels of color change. These findings suggest that viscosity alone may not be a decisive factor in determining the discoloration behavior of CRs. This observation is in line with previous studies that found no significant correlation between viscosity and color change [41,42]. Among all tested materials, Estelite Posterior—a packable CR with an exceptionally high filler content (84% wt/70% vol)—exhibited color stability comparable to that of flowable CRs. These findings are consistent with the results reported by Ardu et al. [34].

Red wine was selected as one of the immersion media in this study due to its well-documented high chromogenic potential and low pH level, approximately 3.0. In conjunction with red wine, this study also included coffee, tea, and cola—beverages that are widely consumed and frequently cited in the literature for their ability to compromise the optical and surface characteristics [36,39,41,42,43]. It is well established that acidic environments can adversely affect the physical and mechanical properties of CRs by degrading the resin–filler interface and facilitating pigment penetration. Within the context of this study, specimens immersed in red wine exhibited the most substantial and statistically significant color changes, followed closely by those exposed to coffee. The pronounced staining effects observed with these two beverages are likely attributable to the combined influence of their acidic pH and polyphenolic content, which can lead to the softening of the matrix and an increase in permeability [35]. Collectively, these factors promote the diffusion of chromogenic agents into the organic matrix of the composites. This phenomenon is further corroborated by the findings of Dietschi et al., who noted the considerable role of hydrophilic environments in facilitating pigment uptake into the polymer network [53]. Interestingly, cola and tea did not result in clinically unacceptable discoloration in most of the tested materials, with the exception of the SDRP group. The relatively lower staining capacity of cola may be attributed to its lack of yellow chromophores, despite its low pH—an observation consistent with findings by previous studies, which reported color change in CRs over time irrespective of immersion medium [32,40,43].

In the present study, following the repolishing procedure, all CRs exhibited varying degrees of color change across the different staining subgroups. While some degree of color recovery was observed in each group, color match to baseline values was not achieved. These findings are consistent with previous studies, which demonstrated that repolishing alone is generally insufficient to fully reverse staining in CRs [41,42]. In support of this, Paolone et al. recently emphasized the importance of repolishing following staining procedures as a conservative intervention to improve aesthetic outcomes [26]. Notably, in the present study, repolishing was effective in restoring color to within clinically acceptable thresholds only for the EUFM, with the exception of specimens immersed in red wine. For SDRP—as well as for all other tested materials subjected to red wine and coffee staining (excluding EUFM)—the repolishing procedure failed to achieve adequate color recovery. These findings suggest that the effectiveness of repolishing is not universal, but rather dependent on multiple factors, including material formulation, filler–matrix interactions, type and intensity of staining agent, and whether the discoloration is intrinsic or extrinsic.

This study has certain limitations, the foremost being its in vitro design and the use of standardized specimens with a thickness of 2 mm. It should be acknowledged that increasing the increment thickness in clinical scenarios may result in reduced depth of cure, which could potentially affect the degree of polymerization and, consequently, the color stability of the CRs. Therefore, further in vivo studies are essential to validate these findings under clinically relevant conditions.

## 5. Conclusions

Within the limitations of the present study, the observed ∆E values were influenced by both the properties of the composite resins and the type of immersing solution. The extent of discoloration varied depending on the brand-specific formulation and compositional differences among the tested materials.

Overall, the results of this study indicate that:

- Red wine and coffee exhibit the highest staining potential, with red wine causing the most profound discoloration across all CRs.
- SDRP was consistently the most susceptible to color change in almost all immersed solutions, whereas EUFM demonstrated superior resistance, particularly in water immersion.
- The influence of filler content, monomer composition, and potential formulation-specific factors of filler may contribute to the tested CR differences.

## Figures and Tables

**Table 1:** Materials and composition of the tested CRs.

Material, Manufacturer, Lot Number	Content	The Filler Weight/Volume%
Spectra STHV SSTHV Dentsply, Milford, DE, USA Lot: 0593	Urethane-modified Bis-GMA; TEGDMA Pre-polymerized SphereTEC fillers, non-agglomerated barium glass, ytterbium fluoride.	78–80/60–62
SDR Plus SDRP Dentsply, Milford, DE, USA Lot: 6955	Modified urethane dimethacrylate resin, EBPADMA, TEGDMA, Camphorquinone, Photoinitiator, BHT, UV Stabilizer, Titanium dioxide, Iron oxide pigments, Barium-alumino-fluoro-borosilicate glass, Strontium alumino-fluoro-silicate glass	70.5/47.4
Estelite Universal Flow Medium EUFM Tokuyama Dental Co., Tokyo, Japan Lot: 1545	Bis-GMA, Bis-MPEPP, TEGDMA, UDMA, Spherical silica-zirconia filler, Camphorquinone, Radical amplifying agent	71/57
Estelite Bulk-Fill Flow EBF Tokuyama Dental Co., Tokyo, Japan Lot: 0784	Bis-GMA, TEGDMA, Bis-MPEPP, Mequinol, Dibutyl hydroxyl toluene and UV absorber, Spherical silica-zirconia filler	70/56
Estelite Posterior EP Tokuyama Dental Co., Tokyo, Japan Lot: W2171	Bis-GMA, TEGDMA, Bis-MPEPP, Spherical silica-zirconia filler	84/70

**Table 2:** The analysis of composite resin and solution factors on ∆E.

	∆E_1_		∆E_2_		∆E_3_	
	F	*p*	F	*p*	F	*p*
Composite resin	12.084	0.001 *	12.393	0.001 *	18.391	0.001 *
Solution	600.069	0.001 *	69.163	0.001 *	155.005	0.001 *
Composite resin * Solution	6.609	0.001 *	9.769	0.001 *	6.036	0.001 *

Two-way ANOVA Test * *p* < 0.05.

**Table 3:** Evaluation of ∆E_1_ according to composite resin and solution.

	SSTHV	SDRP	EUFM	EBF	EP	
Solution	Mean ± SD	Mean ± SD	Mean ± SD	Mean ± SD	Mean ± SD	*p*
Coffee	6.76 ± 1.75 ^Aa^	15.18 ± 1.79 ^Ba^	6.56 ± 0.74 ^Aa^	8.89 ± 1.0 ^Aa^	7.01 ± 1.21 ^Aa^	0.001 *
Tea	2.3 ± 0.45 ^Aa^	8.79 ± 2.34 ^Bb^	1.35 ± 0.55 ^Ab^	1.96 ± 0.85 ^Ab^	2.71 ± 0.49 ^Ab^	0.001 *
Cola	1.3 ± 0.59 ^Aa^	5.59 ± 2.53 ^Bbd^	1.57 ± 0.51 ^Ab^	1.48 ± 0.92 ^Ab^	1.52 ± 0.27 ^Ab^	0.001 *
Wine	30.66 ± 6.72 ^Ab^	22.83 ± 3.01 ^Ac^	24.98 ± 1.02 ^Ac^	27.79 ± 3.63 ^Ac^	26.99 ± 4.32 ^Ac^	0.075
Water	2.19 ± 1.35 ^Aa^	2.52 ± 0.99 ^Ad^	0.43 ± 0.2 ^Bb^	1.85 ± 0.55 ^ABb^	1.11 ± 0.64 ^ABb^	0.006 *
*p*	0.001 *	0.001 *	0.001 *	0.001 *	0.001 *	

Two-way ANOVA Test * *p* < 0.05. Different capital letters in rows indicate differences between composite resin groups. Different lowercase letters in columns indicate differences between solutions.

**Table 4:** Evaluation of ∆E_2_ according to composite resin and solution.

	SSTHV	SDRP	EUFM	EBF	EP	
Solution	Mean ± SD	Mean ± SD	Mean ± SD	Mean ± SD	Mean ± SD	*p*
Coffee	3.68 ± 1.33 ^Aa^	8.12 ± 1.69 ^Ba^	4.16 ± 0.46 ^Aab^	5 ± 2.21 ^Aa^	4.6 ± 1.19 ^Aa^	0.001 *
Tea	1.42 ± 1.29 ^Aa^	6.27 ± 1.17 ^Bab^	0.97 ± 0.49 ^Aa^	2.29 ± 1.37 ^Ab^	2.76 ± 2.13 ^Aa^	0.001 *
Cola	1.84 ± 0.94 ^Aa^	6.7 ± 1.63 ^Bab^	0.77 ± 0.39 ^Aa^	1.35 ± 0.51 ^Ab^	1.74 ± 0.79 ^Aa^	0.001 *
Wine	13.65 ± 5.01 ^ACb^	6.24 ± 1.62 ^ABab^	9.28 ± 6.61 ^ABb^	5.2 ± 1.1 ^Ba^	19.74 ± 4.11 ^Cb^	0.001 *
Water	1.58 ± 0.67 ^Aa^	3.9 ± 2.11 ^Bb^	0.49 ± 0.21 ^Aa^	1.52 ± 1.06 ^Ab^	2.04 ± 1.16 ^ABa^	0.005 *
*p*	0.001 *	0.013 *	0.001 *	0.001 *	0.001 *	

Two-way ANOVA Test * *p* < 0.05. Different capital letters in rows indicate differences between composite resin groups. Different lowercase letters in columns indicate differences between solutions.

**Table 5:** Evaluation of ∆E_3_ according to composite resin and solution.

	SSTHV	SDRP	EUFM	EBF	EP	
Solution	Mean ± SD	Mean ± SD	Mean ± SD	Mean ± SD	Mean ± SD	*p*
Coffee	5.73 ± 1.37 ^Aa^	9.83 ± 0.93 ^Ba^	2.56 ± 0.97 ^Aa^	11.29 ± 2.36 ^Ba^	3.49 ± 0.87 ^Aa^	0.001 *
Tea	3.01 ± 1.39 ^Aba^	5.53 ± 2.34 ^Ab^	0.81 ± 0.23 ^Ba^	3.91 ± 2.32 ^ABb^	3.65 ± 1.06 ^Aba^	0.005 *
Cola	2.03 ± 1.08 ^Aa^	4.4 ± 2.06 ^Bb^	1.76 ± 0.72 ^Aa^	1 ± 0.33 ^Ab^	1.71 ± 0.95 ^Aa^	0.002 *
Wine	19.29 ± 6.36 ^Ab^	17.83 ± 1.92 ^Ac^	16.23 ± 7.46 ^Ab^	22.46 ± 0.9 ^Ac^	7.95 ± 3.69 ^Bb^	0.002 *
Water	2.69 ± 1.11 ^Aa^	4.86 ± 1.65 ^Bb^	0.49 ± 0.21 ^Ca^	1.59 ± 0.59 ^ACb^	2.27 ± 1.38 ^ACa^	0.001 *
*p*	0.001 *	0.001 *	0.001 *	0.001 *	0.001 *	

Two-way ANOVA Test * *p* < 0.05. Different capital letters in rows indicate differences between composite resin groups. Different lowercase letters in columns indicate differences between solutions.

## Data Availability

The data presented in this study are available on request from the corresponding author.

## Abbreviations

The following abbreviations are used in this manuscript:

CR	Composite Resin
BisGMA	Bisphenol A glycidyl dimethacrylate
TEGDMA	Triethylene glycol dimethacrylate
BisEMA	Ethoxylated bisphenol A dimethacrylate
Bis-MPEPP	2,2-bis(4-methacryloxy poly-ethoxyphenyl)propane
UDMA	Urethane dimethacrylate
EBPADMA	Ethoxylated bisphenol A dimethacrylate
STHV	Spectra ST HV
EUFM	Estelite Universal Flow Medium
EBF	Estelite Bulk-Fill Flow
SDRP	SDR Plus
EP	Estelite Posterior
